# The COVID-19 pandemic in Russia: Effects on clinicians and mental health services

**DOI:** 10.1192/j.eurpsy.2021.107

**Published:** 2021-08-13

**Authors:** M. Kulygina, G. Kostyuk

**Affiliations:** 1 Scientific And Education Centre, Mental-health clinic No.1 named after N.A. Alekseev, Moscow, Russian Federation; 2 Director, Mental-health clinic No.1 named after N.A. Alekseev, Moscow, Russian Federation

## Abstract

Since COVID-19 was declared a pandemic by the World Health Organization in March 2020, it has had different infection rates across the world. Russia had one of the largest numbers of infected cases during 2020, but with a lower overall fatality rate. Nevertheless, as in other countries, clinical practice within the mental health care system has faced many stresses and challenges. This concerned the need to organize a treatment of COVID-19 in psychiatric hospitals, as well as a transformation of outpatient care, including psychotherapy, which has largely switched to a remote format. To better understand the effects of the pandemic on mental health professionals, a large-scale study has been implemented through the Global Clinical Practice Network, one of the largest professional communities, which includes 969 members from Russia. The study assessed how COVID-19 affected clinical practice and well-being of clinicians. The first of three surveys was launched in June 2020, in six languages including Russian. Over 2,500 global mental health professionals participated in the study, including 205 clinicians from Russia. Current work circumstances, work-related stressors, and use of telehealth were evaluated. In Russia, the data collection period was characterized by generally improvement in the overall pandemic situation. Results to be presented include the proportion of clinicians that continued working, what kinds of services they provided, their well-being strategies, telehealth modalities and areas in which they had particular concerns about assessment, treatment, or monitoring of patients with mental disorders using remote technologies.

**Disclosure:**

No significant relationships.

